# Using a Facebook group to facilitate faculty-student interactions during preclinical medical education: a retrospective survey analysis

**DOI:** 10.1186/s12909-020-02003-w

**Published:** 2020-03-24

**Authors:** David S. Henry, William D. Wessinger, Nikhil K. Meena, Nalin Payakachat, Jerad M. Gardner, Sung W. Rhee

**Affiliations:** 1 Department of Pharmacology and Toxicology, College of Medicine, University of Arkansas for Medical Sciences, 4301 W. Markham St. Little Rock, Little Rock, AR 72205 USA; 2 Department of Internal Medicine, College of Medicine, University of Arkansas for Medical Sciences, Little Rock, AR USA; 3 Division of Pharmaceutical Evaluation and Policy, College of Pharmacy, University of Arkansas for Medical Sciences, Little Rock, AR USA; 4 Department of Pathology, College of Medicine, University of Arkansas for Medical Sciences, Little Rock, AR USA

## Abstract

**Background:**

Strong learner-teacher relationships are associated with more successful learning outcomes. With shortened modular curricula and increased availability of online resources, fostering faculty interaction with preclinical medical students has become more challenging. We sought to enhance learner-teacher relationships by engaging in discussion with preclinical medical students in their own online space.

**Methods:**

We utilized a closed Facebook discussion group, where faculty and students voluntarily joined in informal discussions and shared announcements related to their courses. The closed discussion group allowed only participating students and faculty to see others’ posts within the group. This provided a platform to freely interact within the confines of the group while maintaining privacy for the personal Facebook accounts of both faculty and students. We utilized the discussion group through three separate organ system-based modules for 14 weeks. Afterward, students were asked to complete an anonymous, voluntary online survey about their experience.

**Results:**

94.1% (160/170) of enrolled second-year medical students joined the voluntary FB discussion group. There were 214 posts, 628 comments, and 4166 reactions in this discussion group during the three modules. Of the students in the group, 74.4% (119/160) responded to the online survey. Overall, students strongly agreed that the Facebook discussion group fostered better rapport with faculty, helped content learning, and improved emotional well-being. Also, they felt more comfortable seeking academic help after using the discussion group. They reported a slight preference for Facebook over email as a medium for asking questions, but no preference for either as a medium for distributing announcements. Students overwhelmingly recommended that the discussion group should be continued in future years.

**Conclusion:**

The Facebook discussion group was a free, efficient, and effective method of cultivating the learner-teacher relationship with the preclinical medical students, resulting in reported enhancement of learning and morale.

## Background

The learner-teacher relationship in medical education is important for cultivating successful learning outcomes [1, 2]. Fostering this relationship between preclinical undergraduate medical students and their teaching faculty has become particularly difficult in recent years. First, as medical schools move to a organ-based curriculum, students often interact with faculty only over a short span of several weeks, as opposed to throughout year-long courses in the traditional model of undergraduate medical education [3]. Second, lectures are now video recorded and are available for live streaming or later viewing. The streaming experience is rapidly improving through better audio-video quality and end-user software. As a result, many students perceive that viewing online lectures at their own pace and without time lost during the commute to school increases learning efficiency [4]. Furthermore, the availability of high-quality, third-party online resources, both subscription-based and free, provides alternatives to faculty-led lectures [5, 6]. For many students, the interaction with faculty is now limited to occasional course announcement emails.

In light of the increasing difficulty of forging the learner-teacher relationship, we, a group of teaching faculty at the University of Arkansas for Medical Sciences (UAMS), searched for ways to increase direct interaction with our second-year undergraduate medical students and to cultivate an inclusive learning environment. Among medical students, Facebook (FB) is the most-used social networking platform, and medical students have found that peer FB groups can be educationally and emotionally supportive [7, 8]. Undergraduate medical students at UAMS also use FB discussion groups for sharing information and resources among peers (Supplementary Figure 1). However, the potential for enhanced interaction and rapport between faculty and students through FB discussion groups in undergraduate medical education has not been explored previously. Several pilot studies in medical or other health science education have shown potential for utilizing social media to facilitate peer-to-peer teaching [8, 9], encourage content discussion [9–12], deliver formative assessments [13–16], and supplement content delivery [14, 15, 17, 18]. To our knowledge, no study has explored the potential of closed FB discussion group for enhancing learner-teacher relationship or rapport. In the present study, we aimed to evaluate students’ perception of the effectiveness of a voluntary, shared faculty-student FB discussion group on fostering learner-teacher relationship and subsequently improving content learning and student morale.

## Methods

At the beginning of the 2016–17 academic year, the two co-directors for the Musculoskeletal and Skin Module (WDW and JMG) created a FB discussion group that was accessible only to second-year medical students and module teaching faculty at UAMS. The discussion group was described as an environment “for students to ask questions and for faculty to respond with clarifications” and where “faculty and students can get to know one another better” (Supplementary Figure 2). Faculty encouraged students to join and participate in the group during lecture and by email. Participation in the discussion group was voluntary and no course points were given. Any new information posted on FB group was repeated as official email to all students. The director for the next Cardiovascular Module (SWR) then joined the discussion group after independently researching forums to answer student questions interactively. After that, the director of the third Pulmonary Module (NKM) continued using the discussion group. During these three modules, we circulated formal module announcements and facilitated informal discussions through the FB group. Due to student demand, we partnered with other teaching faculty to keep the FB discussion group active for the remainder of the academic year.

After the three modules, we solicited student participation via email to a voluntary, anonymous, online questionnaire using the website SurveyMonkey (San Mateo, CA, https://www.surveymonkey.com). The questionnaire contained multiple-choice questions categorizing each student’s frequency and level of use: whether they participated by posting, made comments to posts from others, and/or “liked” or otherwise reacted to posts. We also asked nine electronic visual analog scale (VAS) questions about each student’s experiences with the FB discussion group, and two general open-response questions (Supplementary Figure 3). For the VAS questions, respondents answered by sliding a cursor representing his/her opinion along a continuous interval from one option to another (e.g. “strongly disagree” to “strongly agree”) and each response (from 0 to 200, respectively) was quantified based on the final position of the cursor along the scale [19]. The first five VAS questions (Q2-Q6) were intended to measure the perceived value of using the FB group and showed good internal consistency (Cronbach’s alpha = 0.91) and presented a single factor model (comparative fit = 0.99) [20]. We analyzed the data from these questions (Supplementary Data 1) both in the aggregate (All) and in subgroups divided by use frequency (Daily, Weekly, Rarely). We used one sample t-tests for deviation from 100 (neutral answer) and one-way ANOVA for between-group comparisons using GraphPad Prism (GraphPad Software Inc., La Jolla, CA).

For two open-response questions, two authors (DSH and SWR) independently applied thematic analysis to deduce sets of codes and recurring themes [21]. After consolidating minor differences in the nearly identical sets, each again independently tagged all open responses with one or more codes. During the final meeting, two authors compared the coding of responses and discussed all differences until full agreement was reached (Supplementary Data 2).

## Results

**Fig. 1:**
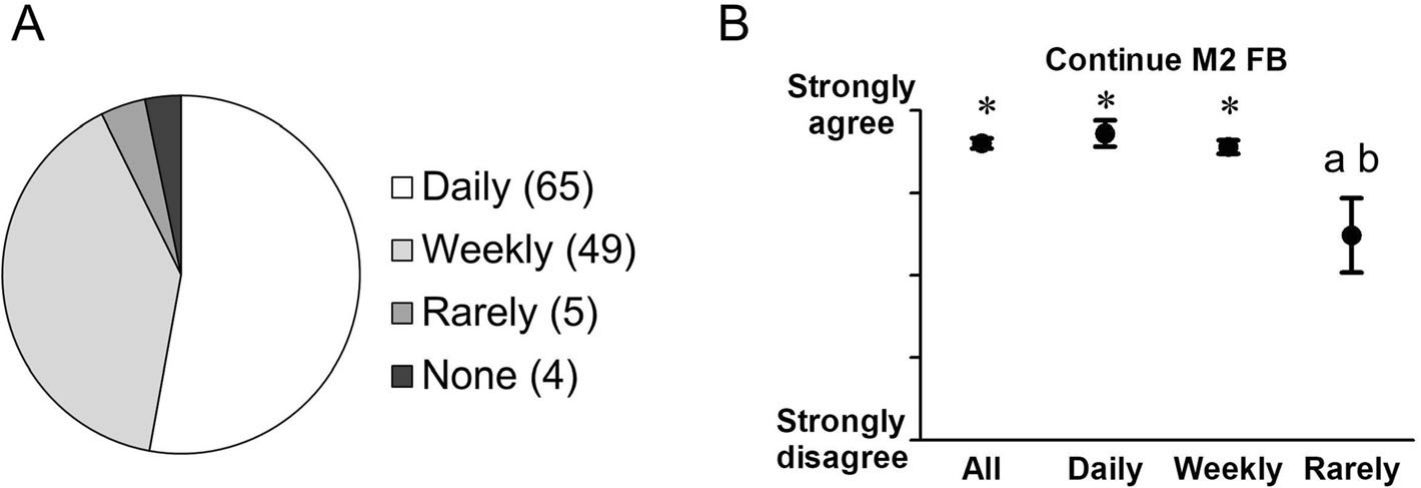
**a** Student responses to voluntary visual analog scale survey regarding their frequency of use of the faculty-student Facebook (FB) discussion group. Survey response rate was 72.4% (123/170). 119 respondents used the group at varying frequencies: Daily, Weekly, or and Rarely. There were 4 students who reported that they did not use the FB group; they were not asked any further questions. **b** Student responses to voluntary visual analog scale survey on whether the faculty-student FB group (M2 FB) should be used for future medical school cohorts. Results were analyzed for all (All) students, as well as by self-reported discussion group use frequency (Daily, Weekly, and Rarely). Error bars depict 95% confidence interval (CI). *: significant difference from neutral by one sample t-test (*p* < 0.05). a: significant difference from Daily (*p* < 0.05). b: significant difference from Weekly (*p* < 0.05)

There were 170 second-year medical students enrolled at UAMS for 2016–17 academic year. Nearly all, or 160 (94.1%), joined the FB discussion group. A large proportion of all students, 123 (72.4%), completed the online questionnaire. Of these respondents, 119 (96.7%) had used the discussion group. When asked how often they used the discussion group (Supplementary Figure 3 Question 11), 65 (54.6%) reported using it daily, 49 (41.2%) reported using weekly, and 5 (4.2%) used the group less often than once a week (denoted “Rarely” in figures and henceforth in the text). Four respondents reported that they did not use the discussion group (i.e., that they were not a member of the discussion group, Supplementary Figure 3 Question 1; “None”). Data for the number of students in each use frequency category are shown in Fig. 1a. Subsequent questions (Table 1 and Supplementary Figure 3) were only asked to the 119 respondents who used the discussion group.

**Table 1:** Survey questions, visual analog scale response options, and student responses to survey questions

Question	Scale			Mean (95%CI)			
	0	100	200	All (*n* = 119)	Daily (*n* = 65)	Weekly (*n* = 49)	Rarely (*n* = 5)
2. M2 FB group improved my rapport/ relationship with the module directors.	Strongly disagree		Strongly agree	169.7* (162.9–176.5)	174.2* (165.0–183.4)	169.1* (159.4–178.7)	106.4 (66.0–146.8)
3. I felt more comfortable seeking help after using the M2 FB group.	Strongly disagree		Strongly agree	155.6* (148.1–163.0)	162.5* (152.2–172.9)	151.3* (140.8–161.8)	106.4 (63.9–148.9)
4. M2 FB group positively contributed to content learning.	Strongly disagree		Strongly agree	166.6* (158.5–172.2)	174.7* (166.1–183.3)	158.5* (148.0–169.0)	112.2 (66.9–157.5)
5. M2 FB group positively contributed to my emotional well-being.	Strongly disagree		Strongly agree	154.2* (146.7–161.7)	161.0* (150.5–171.5)	149.6* (138.6–160.5)	110.8 (65.5–156.1)
6. I would recommend continued use of M2 FB group.	Strongly disagree		Strongly agree	180.3* (174.3–186.4)	186.3* (178.4–194.1)	178.2* (169.9–186.5)	124.4 (61.5–187.3)
7. Asking questions to faculty is easier with:	Email	Equal	Facebook	117.8* (107.0–128.6)	128.1* (112.8–143.4)	112.9 (98.1–127.7)	31.4* (4.4–58.44)
8. Official course announcements are more effective with:	Email	Equal	Facebook	98.3 (85.9–110.8)	109.2 (91.7–126.8)	91.9 (74.0–109.9)	18.8* (− 26.2–63.8)
9. The posts by faculty in M2 FB group were:	Too few	Just right	Too many	100.3 (96.8–103.9)	101.5 (98.3–104.7)	97.0 (89.9–104.1)	118.0 (81.8–154.2)
10. Being Facebook friends with faculty is:	Never appropriate	Neutral	Always appropriate	143.9* (136.9–150.9)	152.0* (142.6–161.5)	137.2* (126.4–147.9)	104.6 (81.2–128.0)

Respondents were enrolled as second-year undergraduate medical students at the University of Arkansas for Medical Sciences during the 2016–2017 academic year. Student responses were analyzed in the aggregate (All) and by Facebook group use frequency (Daily, Weekly, and Rarely). “M2 FB group” refers to the Facebook (FB) discussion group that was available to second-year medical students (M2). *: Significant difference from neutral (100), *p* < 0.05

By the time the questionnaire/survey was conducted, there were 214 posts, 628 comments, and 4166 reactions made by faculty and students in the discussion group. Faculty initiated 80% of the group posts (171/214), including 61 social/fun/encouragement posts, 58 course announcements, and 52 content discussions. Students initiated 20% of posts (43/214), including 14 social/fun posts, 9 announcement or non-content discussions, and 20 content questions. (For examples of each type of posts, see Supplementary Figure 4.) Out of 119 respondents, 24 (20.2%) students reported writing a post in the discussion group, 58 (48.7%) wrote comments to posts, and 111 (93.3%) “liked” or “reacted” to posts made by others (Supplementary Figure 3 Question 12). The most “liked” post from a student was seen by 149 users and received 90 reactions.

All other self-reported data from the questionnaire are presented for the entire group (All) and according to their use frequency categories (Daily, Weekly or Rarely). When asked if they would recommend continued use of the discussion group (Fig. 1b; Table 1 Question 6) the aggregate of students (All), as well as students who reported being Daily, and Weekly users overwhelmingly recommended continued use of the discussion group. Students who used the discussion group Rarely neither agreed nor disagreed that the discussion group should be continued.

**Fig. 2:**
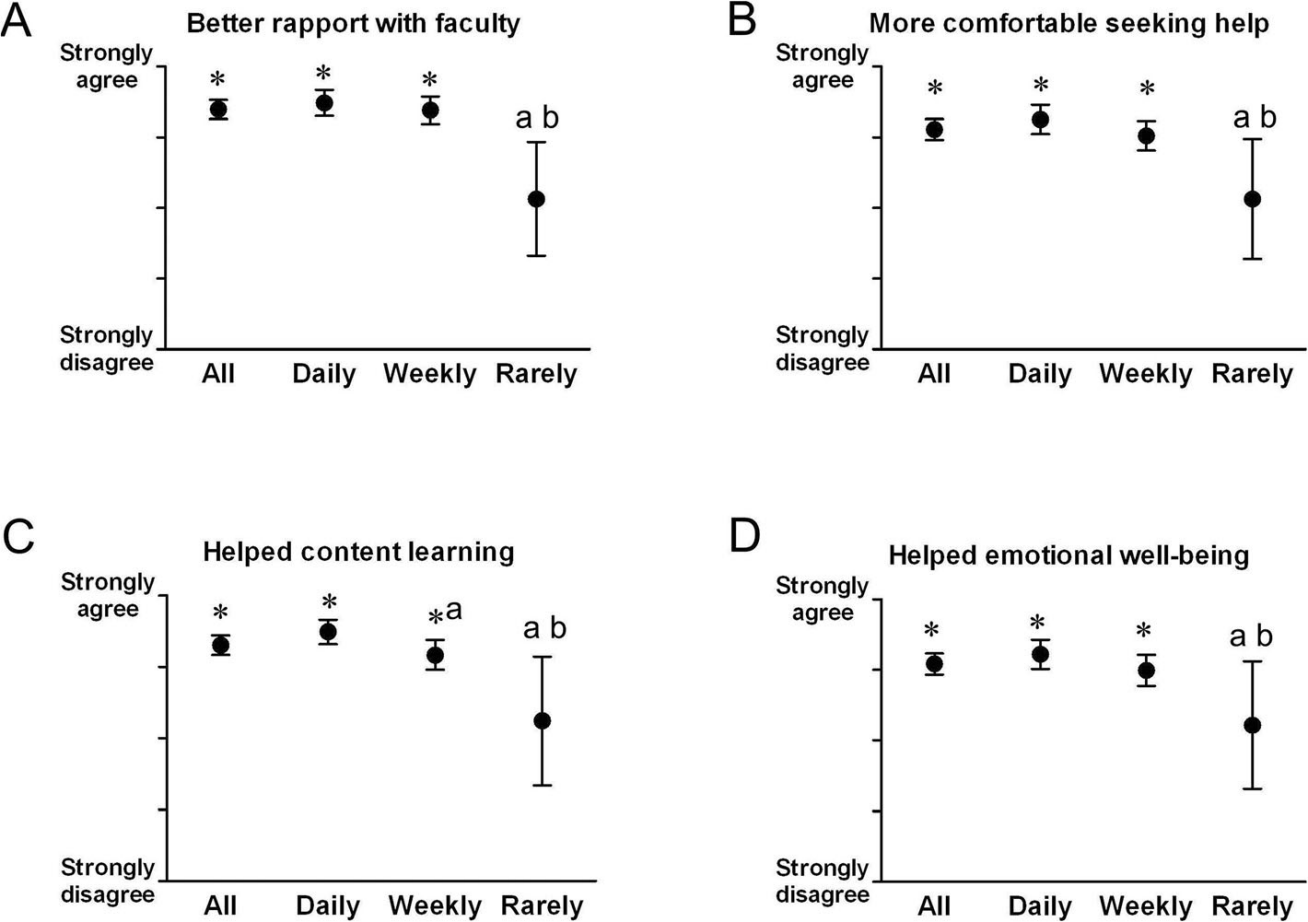
Student responses to visual analog scale survey regarding whether **a**) the M2 FB discussion group built better rapport with faculty, **b**) students felt more comfortable seeking help after using the M2 FB discussion group, **c**) the M2 FB discussion group helped content learning, and **d**) the M2 FB discussion group helped students’ emotional well-being. Results were analyzed for all students (All, *n* = 119), as well as by self-reported discussion group use frequency (Daily, *n* = 65; Weekly, *n* = 49; Rarely, *n* = 5). Error bars depict 95% CI. *: significant difference from neutral by one sample t-test (*p* < 0.05). a: significant difference from Daily (*p* < 0.05). b: significant difference from Weekly (*p* < 0.05)

Overall, students reported that the FB discussion group improved their rapport/relationship with faculty (Fig. 2a; Table 1 Question 2), made them more comfortable seeking help (Fig. 2b; Table 1 Question 3), positively contributed to content learning (Fig. 2c; Table 1 Question 4), and positively contributed to their emotional well-being (Fig. 2d; Table 1 Question 5). When broken down into use frequency categories, both Daily and Weekly users strongly agreed with all four questions: better rapport with faculty, more comfortable seeking help, helped content learning, and helped emotional well-being (Fig. 2; Daily and Weekly). In contrast, students who Rarely used the discussion group were neutral about these questions (Fig. 2; Rarely).

**Fig. 3:**
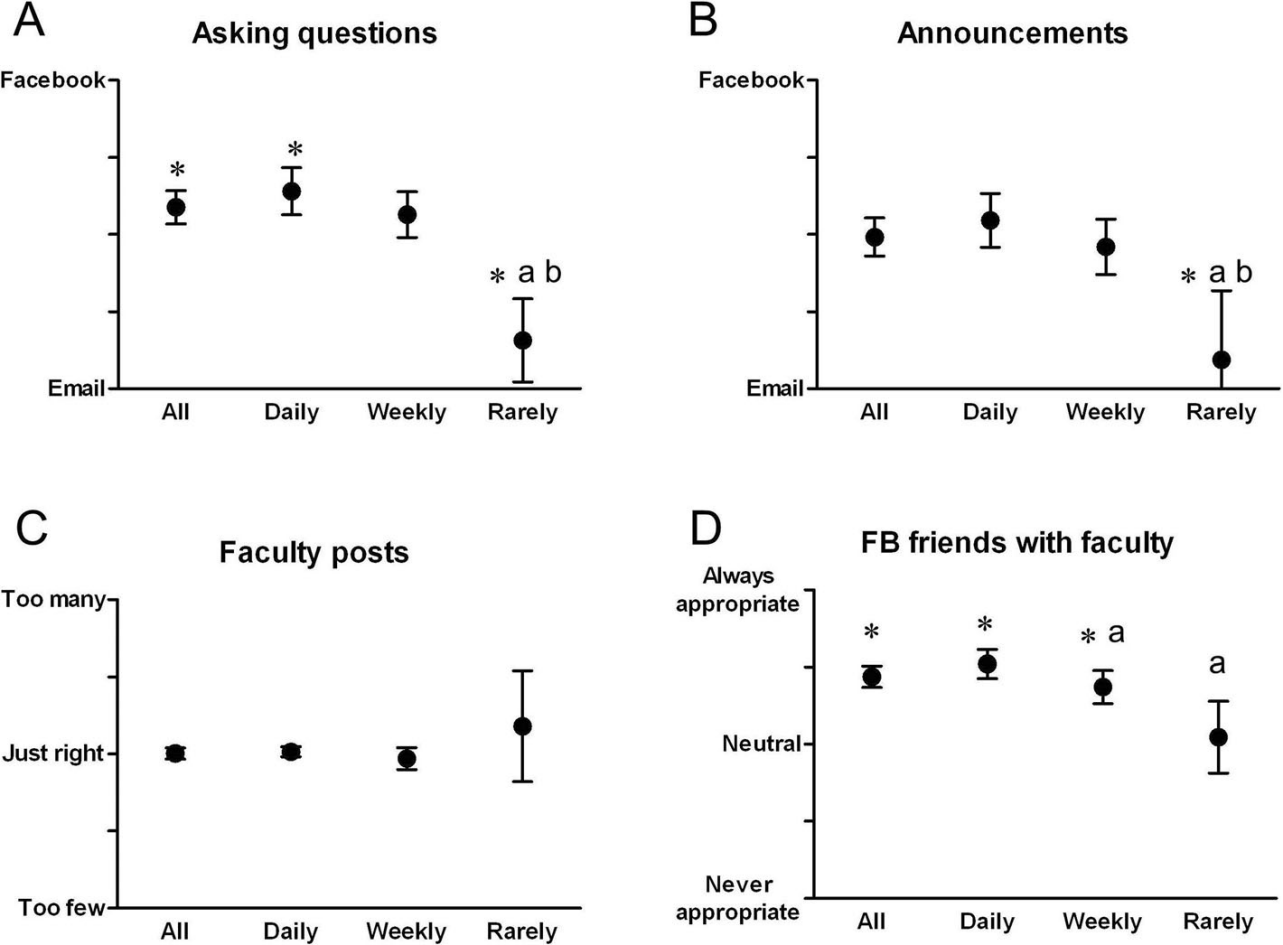
Student responses to visual analog scale survey regarding whether **a**) asking questions to faculty is easier with FB or email, **b**) official course announcements are more effective when sent with FB or email, **c**) there were too many, just right, or too few posts to the FB discussion group by faculty, and **d**) it is appropriate to be FB friends with faculty. Results were analyzed for all students (All, *n* = 119), as well as by self-reported discussion group use frequency (Daily, *n* = 65; Weekly, *n* = 49; Rarely, *n* = 5). Error bars depict 95% CI. *: significantly different from neutral (*p* < 0.05). a: significant difference from Daily (*p* < 0.05). b: significant difference from Weekly (*p* < 0.05)

Overall, students indicated a small preference to FB over email when asking questions to faculty (Fig. 3a All; Table 1 Question 7). By use frequency categories, Daily users answered that FB was easier, Weekly users answered that neither was easier, and students who Rarely used the group answered that email was easier (Fig. 3a). On the question of whether the FB discussion group or email was more effective for distributing official course announcements (Fig. 3b; Table 1 Question 8), most students (All, Daily, and Weekly) reported no preference. Students who Rarely used the group answered that email was more effective. When asked whether the number of posts faculty made was “too many,” “just right,” or “too few,” (Fig. 3c; Table 1 Question 9) All students and students in each use frequency category reported that the number of faculty posts was “just right.”

When asked whether it was appropriate for medical students to be friends with faculty on FB, on a scale of “never appropriate” to “neutral” to “always appropriate,” (Question 10, Table 1; Fig. 3d) All students, Daily users, and Weekly users answered between “neutral” and “always appropriate.” Students who Rarely used the discussion group answered neutrally.

Seventy students answered the open-response questions “What did you like about the M2 FB group?” and/or “Any suggestions or specific concerns?” (Note, “M2 FB group” refers to second-year medical student Facebook group.) We identified three themes: 1) rapport with faculty, 2) usability and function, 3) redundancy (Table 2 and Supplementary Data 2). Within the first theme, students most frequently commented that they appreciated the informal, personal communication and enhanced relationships with faculty (*n* = 43). Students also responded that they perceived faculty to be more accessible via FB (*n* = 11). Whereas the first theme concerned student-faculty relationships, the second theme concerned FB as a platform for communication. Students appreciated the FB group as a fast, easy, efficient, and convenient platform (*n* = 19) and the thread-based format that the FB group provided, which was more conducive to discussion than emails (*n* = 16). Students also remarked that adoption of the FB group was easy, because they already regularly used FB (*n* = 6). The most common negative comment was the inconvenience of receiving the redundant course announcements via FB and email (*n* = 4).

**Table 2:** Qualitative analysis of open-response survey questions

Themes	Codes	Frequency
Rapport with faculty	1a) Informal, personal communication and enhanced relationship with faculty	43
	1b) Accessibility of faculty via Facebook	11
Usability and function	2a) Fast, easy, efficient, and convenient platform	19
	2b) Thread-based discussion format	16
	2c) Facebook already used regularly	6
Redundancy	3) Duplication of announcements sent via email	4

## Discussion

### Need for a new venue to foster learner-teacher relationship

The decrease in the amount of face-to-face instruction in today’s medical education weakens the learner-teacher relationship, which is important for students’ academic success [1, 2], especially for students who are struggling to learn the minimum required content within the strict time constraints of the medical school. In fact, better academic performance has been correlated with face-to-face lecture attendance among medical students [22]. Poor academic performance is both a cause and effect of stress [23], and the focus on students’ physical and emotional well-being in medical education is growing [24, 25]. Medical school is undoubtedly a stressful time, given the academic rigor, pressure to succeed, and personal life stressors such as social disconnectedness [26]. Social disconnectedness is associated with both lower academic performance [27] and higher risk of depression [28].

On the other hand, rapport between students and faculty contribute to several positive student outcomes including perceived learning and motivation [29, 30]. Comprehensive or simplified sets of questions have been asked to measure rapport in those studies [29, 30], but in this study we directly asked students to gauge perceived rapport in a single question. Others described this concept as educational alliance or reduction of psychological distance that leads to improved outcomes [31–33]. Overall, fostering social connectedness, particularly rapport between students and faculty in today’s online society, was a main goal of creating the FB discussion group in this study.

### An informal, convenient, and familiar platform

We chose FB as the platform for our discussion group because it allowed a means of interaction that was efficient, familiar, and comfortable for medical students [7, 8]. Other discussion forums offered through online learning platforms in our previous courses simply did not generate any meaningful participation, which in part led to a formal adoption of email as the sole official communication channel at our institution. Similarly, DiVall and Kirwin found that a course FB page was used more than a Blackboard discussion board when both were used in parallel during a third-year pharmacy course [10]. Perhaps the most striking aspect of our study is that a very high percentage of students (94.1%) joined the group even though it was purely voluntary. Similarly, a high percentage of students actively participated in the group: 95.8% of respondents visited the group at least weekly and 93.3% liked/reacted to the posts. Many of the active FB group participants were not regularly attending lectures so the online interaction with the faculty was the main mode of forging learner-teacher relationship. Most respondents strongly agreed that the discussion group helped form better rapport with faculty (Fig. 2a) and that they felt more comfortable seeking help after using the discussion group (Fig. 2b). In our study, the nature of the FB group interaction was informal, highlighted by the social/fun/encouragement posts frequently created by faculty (61/214 posts, example in Supplementary Figure 4a). In fact, the informal, personal communication and enhanced relationships with faculty was the most frequent student comment (*n* = 43 in Table 2). This informality of interaction and the usability of FB were main strengths of the group that likely helped its wide acceptance (Table 2).

Interestingly, as a platform for asking questions, FB was only slightly preferred over official email communication (Fig. 3a). The format of the FB discussion group allows all members to see any question and contribute cooperatively to the discussion. On one hand, this open forum allows a consolidated thread for discussion of a topic, which is readily accessible. On the other hand, some students may feel intimidated or exposed when asking questions in an open forum and may prefer to privately ask questions to faculty by email. Nevertheless, the improved learner-teacher relationship made students feel more comfortable asking questions in general. From the faculty perspective, better rapport with students made it easier to advise students and get a positive response to suggestions.

While all members of a closed FB group can see posts from any other user made within that closed group, they cannot see posts made on the personal FB accounts of other group members unless they are also FB “friends” with those other users. In this way, FB groups allow faculty and students to freely interact within the confines of the group while still providing a privacy barrier between their personal FB accounts. Interestingly, in contrast to an earlier survey of medical school faculty who deemed it rarely or never appropriate for faculty and students to be friends on FB [34], students in the current study answered that it was mostly appropriate to be FB friends with faculty (Fig. 3d). The discrepancy in these findings likely stems from perception of faculty versus students and changes in general attitudes about social media over the past decade.

### Rapport and perceived learning - comparison to other studies

In our review of literature, we did not find other studies among health science education that evaluated the potential of FB groups to improve rapport between faculty and students. Instead, studies mainly focused on the instructional aspect, either as faculty-led or faculty-moderated student interaction [8–18]. In one related study, Alshiekhly et al. reported that 83.7% of students rated the interaction between students and the group administrators as “good” or “excellent” in a FB group designed to teach students about medical emergencies in dental practice [13]. However, only the quality of the ‘teaching’ interaction was assessed instead of the rapport those interactions may have fostered. Irwin et al. cited student comments to suggest that a FB page for several public health courses “enhanced communication and interaction between students and the course instructors,” but, similarly to Alshiekhly et al., did not address how that communication affected rapport or relationships between students and faculty [11].

Medical students perceived overall that our FB group positively contributed to the content learning (Fig. 2c). Several studies focused on the learning aspect of FB groups shared similar conclusions. Pickering and Bickerdike reported that among medical school students taking an anatomy course in the United Kingdom, 85.0% of respondents reported that a faculty-student FB page “was an effective tool in advancing their learning,” and 88.5% responded that it “compared favorably to what was already available.” [12] The design of their site was very similar to ours, an optional, ungraded, supplemental FB page to facilitate faculty-student discussion, without redundancy in course materials otherwise available. Ravindran et al. reported similarly that 94.8% of undergraduate medical students in the clinical phase of their education who participated in a FB teaching group reported that the group “supplemented [their] learning relevant to [their] exam.” [16] However, the primary goal of that group was to increase content learning. As such, content posted by the teaching fellow who facilitated the group was limited to practice questions and discussions of those questions. In these studies, student perception of increased rapport with faculty and emotional well-being were not measured. While our study and the aforementioned studies assess student perceptions of content learning, Anwar et al. reported increased grades among a cohort of undergraduate medical students in a neuroscience course with access to a curated FB page where multiple choice questions, articles, links, and lecture notes were posted [14]. In short, several studies among undergraduate medical students support the potential, either real or perceived, of increased content learning associated with FB group use, and our study concurs.

### Faculty perspective and future directions

There is now a wide body of literature on the use of social media in medicine. Social media platforms allow physicians to network with one another, share medical knowledge, interact with patient groups, advocate on behalf of rare diseases, and provide general education to the non-medical public [35–43]. Among these platforms, FB is the largest and most well-known. At the time our FB discussion group was formed (August 2016), FB use had grown to be ubiquitous, with 1.860 billion active users worldwide (Q4, 2016) [44]. FB groups provide a free and easy-to-use platform for medical education that can be adapted to small local groups like ours all the way up to massive global groups with tens of thousands of members [45]. Our FB group for medical student and faculty interaction was initially created as a pilot project attempting to enhance the medical school experience for learners in our modules. It was such a positive experience both for faculty and for students that we pursued this formal survey evaluation to quantify and better understand student’s perceptions of the group. We are encouraged by these data and plan to continue using similar FB groups in future years. Future directions for study include formally evaluating our medical school faculty regarding their experiences and perceptions of using this FB group as well as evaluating faculty who chose not to participate in the group so as to better understand concerns and potential barriers to faculty participation. A prospective randomized control trial contrasting academic performance of students given the opportunity to participate in a faculty-student FB discussion group with the academic performance of students that are not offered this opportunity would be a potential method to objectively evaluate the educational impact of FB groups like this.

As the general population increasingly utilizes social media to connect with others and to share and consume information, it stands to reason that future medical students will do the same. There are significant differences between personal and professional use of social media, and as medical school faculty, we need to educate our students on how to appropriately and beneficially use social media, not only as students, but also as future practicing physicians. If faculty are not willing to learn how to use social media, and to engage and interact with students in that sphere, will we be able to effectively teach and model proper professional online conduct for our students?

We see the FB discussion group as a natural extension of medical school to a format that is familiar and convenient to the emerging generation of physicians. Even though online interactions may not be face-to-face, they can have a similar emotional and social impact, leading to the development of professional relationships that translate into 'real life' [46, 47]. Medical school faculty who show willingness to adapt to novel methods of teaching and student engagement stand to gain much, not only for the benefit of their students, but also their own academic careers [48, 49].

### Limitations

There are several limitations to this study. First, all measures assessed by the questionnaire were the students’ perceptions of the outcomes, rather than the outcomes themselves. For example, whether the perceived help in content learning actually resulted in better exam score remains unknown. In addition to the new FB discussion group, several other changes were introduced during the 2016–17 academic year including three new module directors (WDW, SWR, and NKM), many new lectures and new in-house exam procedures. Therefore, it remains difficult to pinpoint whether the FB discussion group had a singular positive effect on student performances. Second, student responses may have been subject to biases such as social desirability and acquiescence biases [50]. Student reactions in the FB discussion group may have been influenced by the social desirability bias [51] as the identity of students were not hidden in FB interactions. Students may have wanted to appear in the best light to the faculty and to be supportive of faculty who tried to improve communications with them, rather than express what they actually liked or disliked in the discussion group. However, this social desirability bias should be minimized through the use of an anonymous survey [52]. Acquiescence bias occurs as individuals tend to provide affirmative answers to questions regardless of the content [53]. In order to minimize this bias, we presented questions in a bidirectional fashion with verbal explanations on both ends of the VAS questions [54]. The default slider appeared in the middle (neutral position) for each VAS question and students’ answers were only registered when they actually confirmed the neutral position or moved the position of the slider. Third, although a positive learner-teacher relationship is associated with positive outcomes in medical education [1, 2], whether there are actual causal relationships between better rapport, feeling comfortable seeking help, content learning, and emotional well-being could not be determined in the questionnaire format of this study.

Although the FB discussion group expanded the number of students that faculty could connect with to improve the learner-teacher relationship, the faculty still could not engage all students. Because the group was purely voluntary, students who either did not have FB accounts or did not wish to actively participate in the discussion group were not reached. Unsurprisingly, students’ perceived benefit from use of the discussion group strongly correlated with frequency of use, and we posit that students’ decisions about using a particular resource are chiefly determined by their perceived helpfulness of the resource. Also, because of the voluntary nature of the discussion group, information shared in the group had to be duplicated in the form of official email to all students. This was perceived by some students as information overload and was the most common criticism.

## Conclusions

A closed FB discussion group provided a free and efficient method of communication between the faculty and preclinical medical students. Over 90% of students actively participated in the group through posts, comments, and reactions, cultivating the learner-teacher relationship. Students reported a significant improvement in rapport with faculty, learning of the content material, and general morale as a result of the faculty-student discussion group.

## Supplementary information

**Additional file 1.** Supplementary Figures. Screen captures of Facebook posts and survey items

**Additional file 2.** Supplementary Data 1. Quantitative survey data: spreadsheet of all quantitative survey data

**Additional file 3.** Supplementary Data 2. Open responses and analysis: spreadsheet of all open response answers and qualitative coding

## Data Availability

All data generated or analyzed during this study are included in this published article and its supplementary information files.

## Abbreviations

CI: Confidence Interval
FB: Facebook
UAMS: University of Arkansas for Medical Sciences
VAS: Visual analog scale

## References

[1] Norman GR, van der Vleuten CPM, Newble DI, Tiberius RG, Sinai J, Flak EA (2002). The role of teacher-learner relationships in medical education. International handbook of research in medical education.

[2] Kanter SL (2012). To be there or not to be there: is attendance really the question?. Acad Med.

[3] Takeuchi JS, Smith NM, Mortimer AM (1983). Innovative models of medical education in the United States today: an overview with implications for curriculum and program evaluation. In: medical education and societal needs: a planning report for the health professions.

[4] Cardall S, Krupat E, Ulrich M (2008). Live lecture versus video-recorded lecture: are students voting with their feet?. Acad Med.

[5] Midle JB, Molgaard K, Albright S, Karimbux N (2016). Relationship among dental students’ class lecture attendance, use of online resources, and performance. J Dent Educ.

[6] Gupta A, Saks NS (2013). Exploring medical student decisions regarding attending live lectures and using recorded lectures. Med Teach.

[7] Pander T, Pinilla S, Dimitriadis K, Fischer MR (2014). The use of Facebook in medical education--a literature review. GMS Z Med Ausbild.

[8] Ali A (2016). Medical students’ use of Facebook for educational purposes. Perspect Med Educ.

[9] Estus EL (2010). Using facebook within a geriatric pharmacotherapy course. Am J Pharm Educ.

[10] Divall MV, Kirwin JL (2012). Using facebook to facilitate course-related discussion between students and faculty members. Am J Pharm Educ.

[11] Irwin C, Ball L, Desbrow B, Leveritt M (2012). Students’ perceptions of using Facebook as an interactive learning resource at university. Australas J Educ Technol.

[12] Pickering JD, Bickerdike SR (2017). Medical student use of Facebook to support preparation for anatomy assessments. Anat Sci Educ.

[13] Alshiekhly U, Arrar R, Barngkgei I, Dashash M (2015). Facebook as a learning environment for teaching medical emergencies in dental practice. Educ Heal Chang Learn Pract.

[14] Anwar K, Sajid MR, Cahusac P, Shaikh AA, Elgammal A, Alshedoukhy A (2017). Can Facebook pages be a mode of blended learning to supplement in-class teaching in Saudi Arabia?. Adv Physiol Educ.

[15] Jaffar AA (2014). Exploring the use of a facebook page in anatomy education. Anat Sci Educ.

[16] Ravindran R, Kashyap M, Lilis L, Vivekanantham S, Phoenix G (2014). Evaluation of an online medical teaching forum. Clin Teach.

[17] Cain J, Policastri A (2011). Using facebook as an informal learning environment. Am J Pharm Educ.

[18] Ross JG, Beckmann B, Goumas C (2019). Baccalaureate nursing students’ perceptions of the use of a Facebook case study as a teaching strategy. Nurs Educ Perspect.

[19] Couper MP, Tourangeau R, Conrad FG, Singer E (2006). Evaluating the effectiveness of visual analog scales: a web experiment. Soc Sci Comput Rev.

[20] Carmines EG, Zeller RA (1979). Reliability and validity assessment.

[21] Chapman AL, Hadfield M, Chapman CJ (2015). Qualitative research in healthcare: an introduction to grounded theory using thematic analysis. J R Coll Physicians Edinb.

[22] Abdulghani HM, Al-Drees AA, Khalil MS, Ahmad F, Ponnamperuma GG, Amin Z (2014). What factors determine academic achievement in high achieving undergraduate medical students? A qualitative study. Med Teach..

[23] Stewart SM, Lam TH, Betson CL, Wong CM (1999). Wong a M. a prospective analysis of stress and academic performance in the first two years of medical school. Med Educ.

[24] Wasson LT, Cusmano A, Meli L, Louh I, Falzon L, Hampsey M (2016). Association between learning environment interventions and medical student well-being. JAMA.

[25] Slavin SJ, Schindler DL, Chibnall JT (2014). Medical student mental health 3.0: improving student wellness through curricular changes. Acad Med.

[26] Vitaliano PP, Russo J, Carr JE, Heerwagen JH (1984). Medical school pressures and their relationship to anxiety. J Nerv Ment Dis.

[27] Robbins SB, Le H, Davis D, Lauver K, Langley R, Carlstrom A (2004). Do psychosocial and study skill factors predict college outcomes?. A Meta-Analysis Psychol Bull.

[28] Armstrong S, Oomen-Early J (2009). Social connectedness, self-esteem, and depression symptomatology among collegiate athletes versus nonathletes. J Am Coll Heal.

[29] Lammers WJ, Gillaspy JA (2013). Brief Measure of Student-Instructor Rapport Predicts Student Success in Online Courses. Int J Scholarsh Teach Learn.

[30] Wilson JH, Ryan RG (2013). Professor–student rapport scale: six items predict student outcomes. Teach Psychol.

[31] Baepler P, Walker JD (2014). Active learning classorooms and educational alliances: changing relationships to improve learning. New Dir Teach Learn.

[32] Liberman N, Trope Y, Stephan E, Kruglanski AW, Tory Higgins E (2007). Psychological distance. Social psychology: handbook of basic principles.

[33] Tiberius RG. The why of teacher/student relationships. Essays Teach Excell. 1993–4;5(8):1–5.

[34] Chretien KC, Farnan JM, Greysen SR, Kind T (2011). To friend or not to friend? Social networking and faculty perceptions of online professionalism. Acad Med.

[35] Isom J, Walsh M, Gardner JM (2017). Social media and pathology: where are we now and why does it matter?. Adv Anat Pathol.

[36] Fuller MY, Allen TC (2016). Let’s have a tweetup: the case for using twitter professionally. Arch Pathol Lab Med..

[37] Fuller MY, Mukhopadhyay S, Gardner JM (2016). Using the periscope live video-streaming application for global pathology education: A brief introduction. Arch Pathol Lab Med.

[38] Cohen D, Allen TC, Balci S, Cagle PT, Teruya-Feldstein J, Fine SW (2017). #InSituPathologists: how the #USCAP2015 meeting went viral on twitter and founded the social media movement for the United States and Canadian academy of pathology. Mod Pathol.

[39] Gardner JM (2017). How angiosarcoma and facebook changed my life. Arch Pathol Lab Med.

[40] Carlquist E, Lee NE, Shalin SC, Goodman M, Gardner JM (2017). Dermatopathology and social media: a survey of 131 medical professionals from 29 countries. Arch Pathol Lab Med.

[41] Haller J, David MP, Lee NE, Shalin SC, Gardner JM (2018). Impact of pathologist involvement in sarcoma and rare tumor patient support groups on Facebook: a survey of 542 patients and family members. Arch Pathol Lab Med.

[42] Gottesman SP, Klein WM, Hosler GA, Veprauskas KR, Rush PS, Gardner JM (2018). #dermpathJC: the first online dermatopathology twitter journal club. J Cutan Pathol.

[43] Nix J, Gardner J, Costa F, Soares A, Rodriguez F, Moore B (2018). Neuropathology education using social media. J Neuropathol Exp Neurol.

[44] Facebook Q1 2018 Results [Internet]. Menlo Park; 2018 [cited 2018 Jun 29]. Available from: https://s21.q4cdn.com/399680738/files/doc_financials/2018/Q1/Q1-2018-Earnings-Presentation-(1).pdf.

[45] Gonzalez RS, Amer SM, Ben YN, FDA C, Noatay M, Qiao JH (2017). Facebook discussion groups provide a robust worldwide platform for free pathology education. Archives of Pathology and Laboratory Medicine.

[46] Madke B, Gardner JM (2017). Enhanced worldwide dermatology–pathology interaction via Facebook, twitter, and other social media platforms. Am J Dermatopathol.

[47] Oltulu P, Mannan AASR, Gardner JM (2018). Effective use of twitter and Facebook in pathology practice. Hum Pathol.

[48] Freitag CE, Arnold MA, Gardner JM, Arnold CA (2017). If you are not on social media, here’s what you’re missing! #dothething. Arch Pathol Lab Med..

[49] Gardner JM. Social media evaluation for Jerad Gardner, MD promotion to associate professor. [Internet]. 2016 [cited 2018 Jun 28]. Available from: https://www.surveymonkey.com/results/SM-VRPSJKLM/.

[50] Alreck PL, Settle RB (2004). The survey research handbook.

[51] Nancarrow C, Brace I (2000). Saying the “right thing”: coping with social desirability bias in marketing research. Bristol Bus Sch Teach Res Rev.

[52] Paulhus DL, Reid DB (1991). Enhancement and denial in socially desirable responding. J Pers Soc Psychol [Internet].

[53] Messick S. The psychology of acquiescence: An interpretation of research evidence. ETS Res Bull Ser. 1966:i–44. 10.1002/j.2333-8504.1966.tb00357.x.

[54] Hinz A, Michalski D, Schwarz R, Herzberg PY (2007). The acquiescence effect in responding to a questionnaire. Psychosoc Med.

