# Supplementary material for: Using a Facebook group to facilitate faculty-student interactions during preclinical medical education: a retrospective survey analysis
Source: BMC Med Educ. 2020 Mar 24;20:87. doi: 10.1186/s12909-020-02003-w (PMC7092445; doi:10.1186/s12909-020-02003-w)
Supplement: Supplementary file 1 — Additional file 1. Supplementary Figures. Screen captures of Facebook posts and survey items [file 12909_2020_2003_MOESM1_ESM.pdf]

Supplementary Figure 1. Search result for Facebook student discussion groups in the College of Medicine (COM) at the University of Arkansas for Medical Sciences (UAMS)

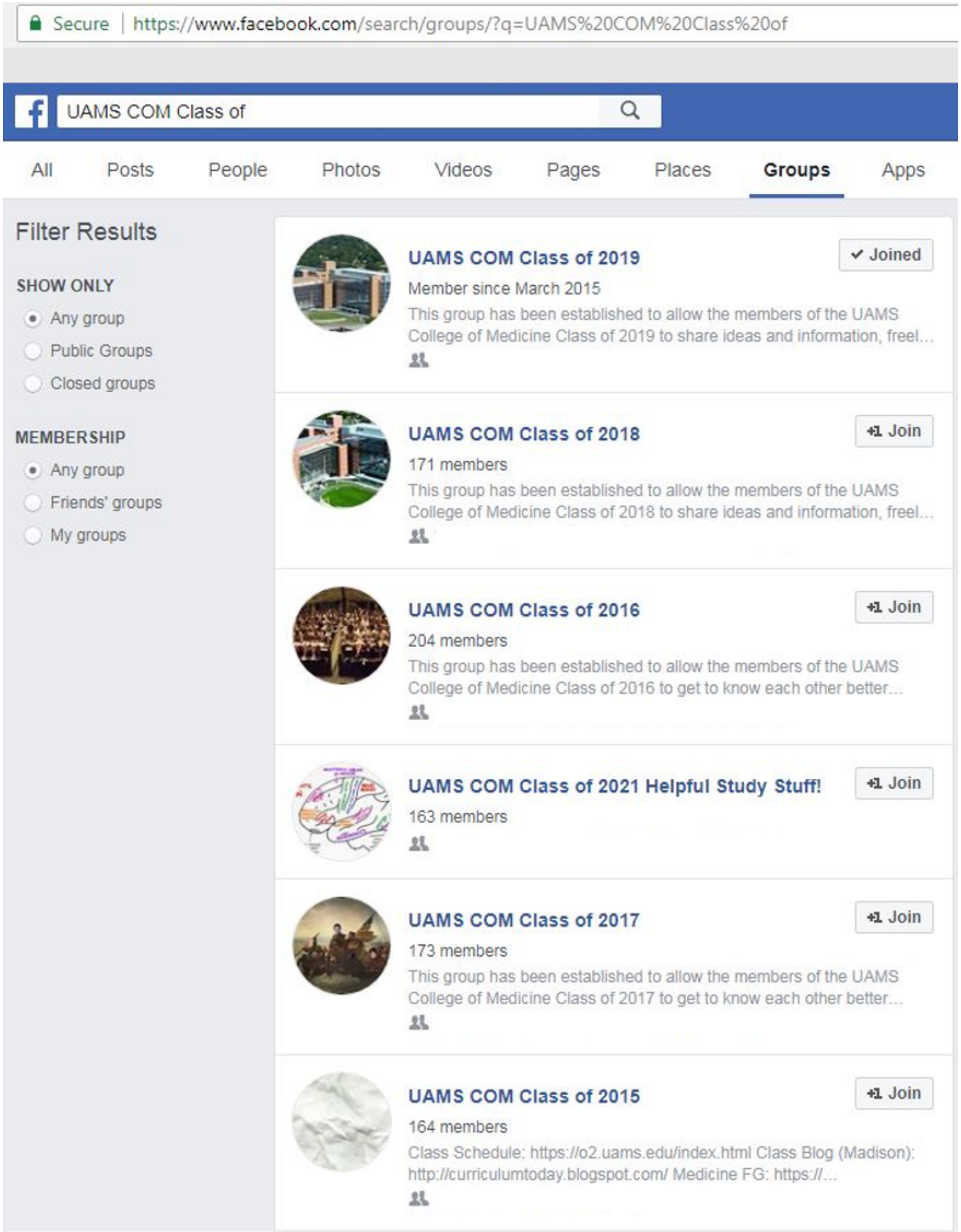

Supplementary Figure 2. Description of the Facebook discussion group

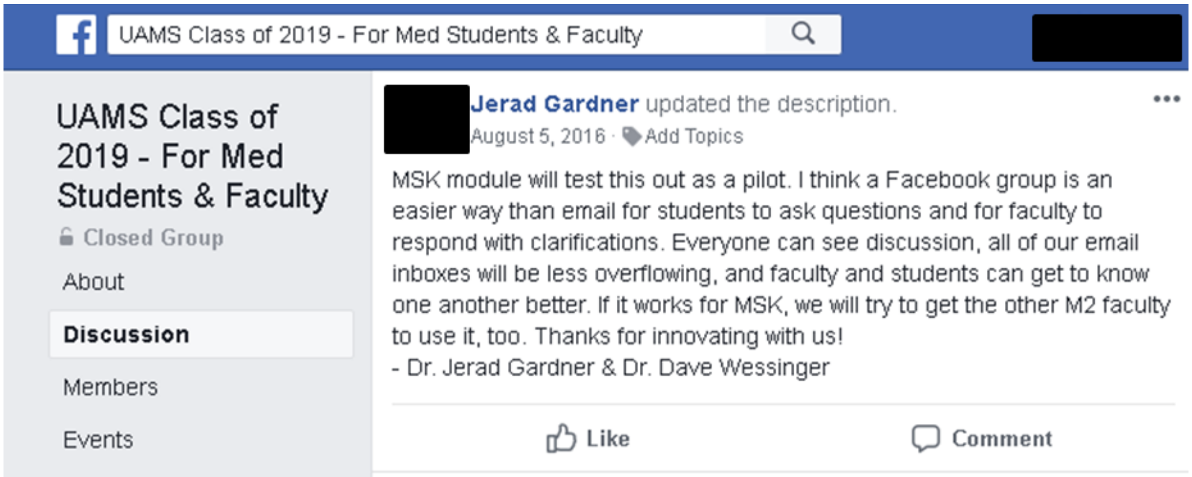

Supplementary Figure 3. Survey questions and answer choices.

1. Are you a member of the UAMS M2 FB group?

- Yes
- No

2. M2 FB group improved my rapport/relationship with the module directors.

Strongly disagree — Strongly agree

3. I felt more comfortable seeking help after using M2 FB group.

Strongly disagree — Strongly agree

4. M2 FB group positively contributed to content learning.

Strongly disagree — Strongly agree

5. M2 FB group positively contributed to my emotional well-being.

Strongly disagree — Strongly agree

6. I would recommend continued use of M2 FB group.

Strongly disagree — Strongly agree

7. Asking questions to faculty is easier with:

Email — Equal — Facebook

8. Official course announcements are more effective with:

Email — Equal — Facebook

9. The posts by faculty in M2 FB group were:

Too few — Just right — Too many

10. Being Facebook friends with faculty is:

Never appropriate — Neutral — Always appropriate

11. How often did you use M2 FB group?

- ◯ Daily
- ◯ Weekly
- ◯ Less often than once a week

12. Please mark your activity in M2 FB group. (Choose all that apply.)

- ☐ Posted
- ☐ Commented
- ☐ Liked or reacted

13. What did you like about the M2 FB group?

14. Any suggestions or specific concerns?

Supplementary Figure 4. Examples of type of FB posts

a. Social/fun/encouragement post

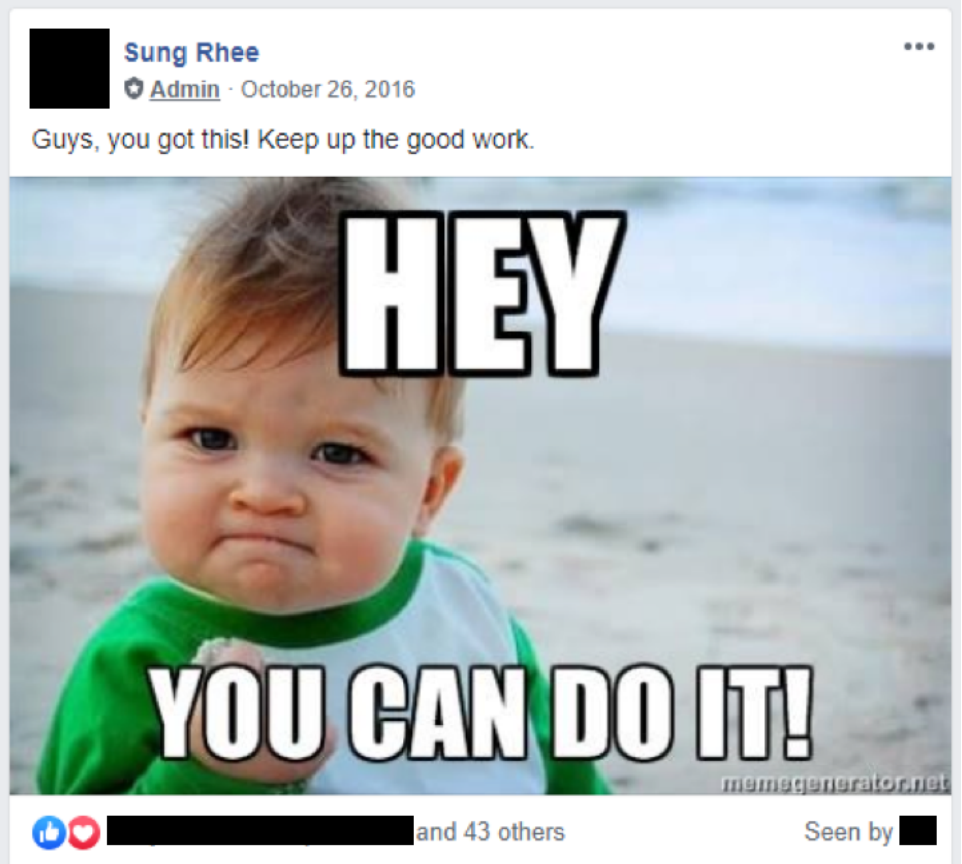

b. Course announcement

[redacted]
Admin · September 5, 2016

M2 CV checklist for tomorrow. Prizes for active participation!
1. Plan on attending lectures tomorrow, starting at 8:30 am. Recordings may not work.
2. Bring clickers if you have one.
3. Textbooks: Lilly, BRS Physiology, FirstAid, (Katzung)
4. M2 CV on Blackboard: http://tinyurl.com/zdprpm9... See More

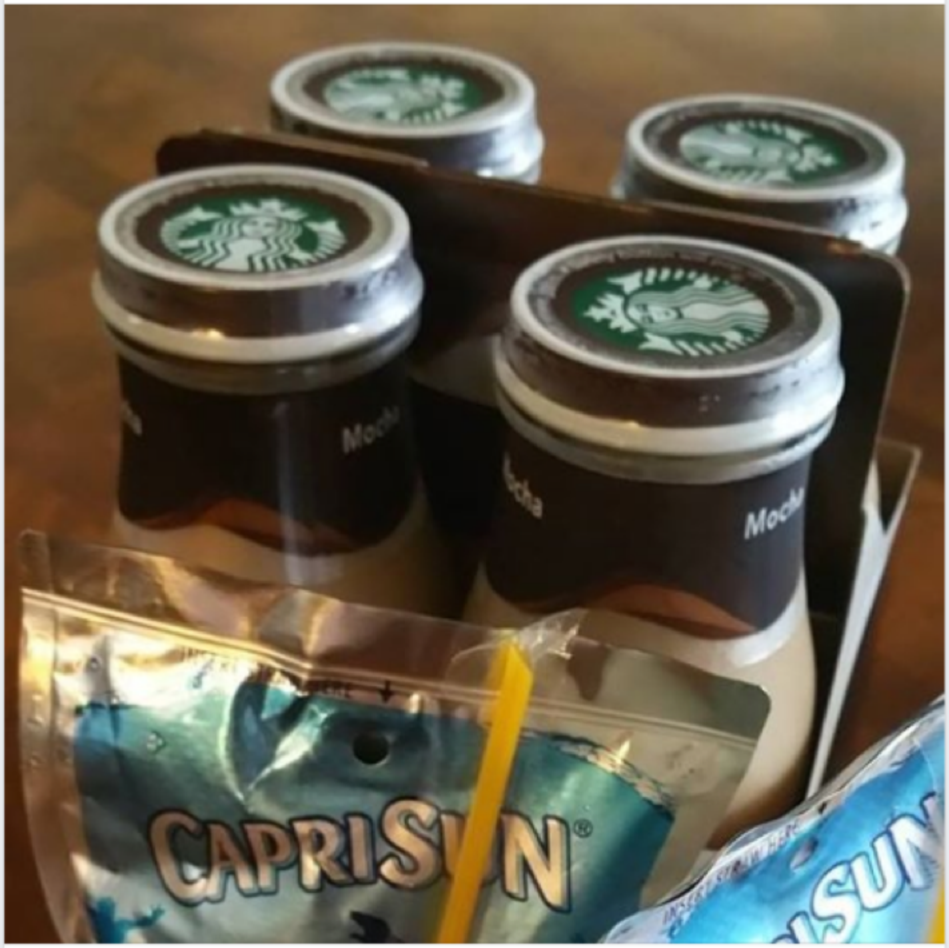

[redacted] and 9 others 3 Comments Seen by [redacted]

Like Comment

[redacted] I'm guessing Starbucks and Capri Sun is the key to success.
Like · Reply · 3y 3

Sung Rhee Starbucks yes. Capri sun not so much.
Like · Reply · 3y 9

c. Faculty-initiated content discussion

Sung Rhee
Admin · September 16, 2016

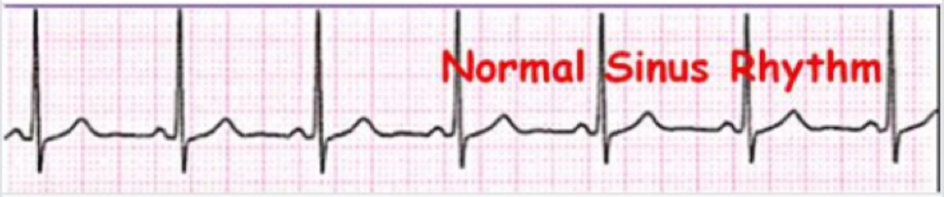

[redacted] and 1 other · 7 Comments · Seen by [redacted]

Like · Comment

Sung Rhee Now that you got the lecture, what is the rate for this ECG?
Like · Reply · 3y

> [redacted] ~ 75 bpm
> Like · Reply · 3y
>
> **Sung Rhee** Pretty close to that. Slightly slower or faster?
> Like · Reply · 3y
>
> [redacted] A little faster, looks like.
> Like · Reply · 3y
>
> [redacted] I got 79bpm, depending on how good your eyes are
> Like · Reply · 3y
>
> [redacted] Mine aren't the best. Anyone going into optho? Looks like I'll need it
> Like · Reply · 3y · Edited · 1
>
> **Sung Rhee** You just need an approximate value on multiple choice exams
> Like · Reply · 3y · 3

d. Student-initiated content discussion

[redacted]
September 15, 2016

This may be a silly question but it is confusing me. So in the lecture today on Antihypertensive drugs, when discussing the Angiotensin II receptor blockers (ARBs) the slide says the blockers bind to ATI receptors to prevent binding of AT II. Wouldn't ATI receptors bind ATI and wouldn't you need AT II receptors for ATII, I could be thinking about this all wrong but its really messing with my head. Even the title on slide 32 says Angiotensin II receptor blocker, but then it goes on to say the blocker binds to AT1

[redacted] 4 Comments Seen by [redacted]

Like Comment

[redacted] I believe it stands for Angiotensin Type 1 receptor
Like · Reply · 3y

[redacted] AT1 is an angiotensin II receptor (subtype 1)
https://en.wikipedia.org/wiki/Angiotensin_II_receptor_type_1

EN.WIKIPEDIA.ORG
Angiotensin II receptor type 1 - Wikipedia, the free encyclopedia

1
Like · Reply · 3y

[redacted] ohhh ok I knew something must be off, I was reading it as AT1=angiotensin 1, when AT1= Angiotensin2 receptor type 1. Ok thanks I understand now. I still think that's a confusing way of saying it.
2
Like · Reply · 3y

Sung Rhee To avoid confusion, I suggest using AT1R and AngII. There is also a receptor called AT2R (not covered in CV module). AngII binds to both AT1R and AT2R.
Like · Reply · 3y
